# Growth and yield of greenhouse eggplant under extended photoperiods using light emitting diodes

**DOI:** 10.3389/fpls.2025.1737061

**Published:** 2026-01-19

**Authors:** Daniel Terlizzese, Jason Lanoue, Celeste Little, Sarah St. Louis, Youbin Zheng, Xiuming Hao

**Affiliations:** 1 School of Environmental Sciences, University of Guelph, Guelph, ON, Canada; 2 Harrow Research and Development Centre, Agriculture & Agri-Food Canada, Harrow, ON, Canada

**Keywords:** biomass allocation, blue light, chlorophyll fluorescence, continuous light, *Solanum melongena*, supplemental light

## Abstract

Supplemental lighting can significantly increase production of greenhouse vegetable crops during seasons of low natural light, but the cost of electricity to support supplemental lighting often poses a substantial burden on greenhouse growers. Electrical costs can be mitigated, while maintaining the same daily light integral (DLI), by decreasing the light intensity and extending the supplemental photoperiod. We provided greenhouse eggplant (*Solanum melongena*) cv. Jaylo with three distinctive supplemental light photoperiods: 16-hour of white light, 20-hour of white light, and 16-hour of white light followed by 8h of blue light [24-hour continuous lighting (CL)]. Each photoperiod treatment had a supplemental DLI of 8.64 mol day^-1^ and was compared to an unlit control group which only had natural light. No significant difference in fruit yield was found between any of the lit treatments, but supplemental lighting treatments increased total yield by 124-144% relative to the unlit control. A 20-hour photoperiod contributed to elevated levels of leaf stress early in the trial, recorded through dark-adapted and light-adapted chlorophyll fluorescence metrics, while plants treated with 16-hour of supplemental light achieved the greatest vegetative biomass. However, leaf stress was not recorded in the 20-hour treatment plants as the trial progressed, as the natural light DLI rose. Our data illustrated that a CL strategy resulted in similar yields and comparable plant growth as other supplemental photoperiods; therefore, we implore greenhouse eggplant growers to utilize a dynamic 24-hour photoperiod to possibly reduce the electrical cost of supplemental lighting during the winter.

## 1 Introduction

Vegetable crop yield in greenhouses located in northern high latitudes is vastly reduced during winter months due to the lack of sun energy available to plants. The use of electrical lighting to supplement periods of low natural light during the winter season has proven to be effective at improving crop growth and yield (Hao et al., 2018; Lanoue et al., 2018; Olle and Viršile, 2013). Supplemental lighting has therefore become essential for many greenhouses to maintain production of fruit and vegetables and meet the demand of consumers. High-intensity discharge (HID) and light emitting diodes (LED) are the most popular lighting types used among high output supplemental lighting fixtures. However, LEDs are becoming more attractive for horticultural applications due to their higher photosynthetic photon efficacy (PPE), dynamic spectral capabilities, and dimmability (Nelson and Bugbee, 2014).

Although supplemental lighting has the potential to increase crop production, the increased demand for light fixture capital investment and electricity can account for a large percentage of greenhouse production costs (van Iersel and Gianino, 2017; Watson et al., 2018) and may deter greenhouse growers from utilizing supplemental lights. Therefore, research focused on the optimal use of supplemental lighting to increase light use efficiency (LUE) and decrease the light fixture cost and electrical cost for greenhouse vegetable production is critical.

Longer photoperiods have been proposed as a strategy to increase LUE and decrease operating costs of supplemental lighting (Aguirre-Becerra et al., 2020; Weaver et al. 2019). In general, photosynthetic efficiency (moles of carbon fixed per photon) decreases as light intensity increases (Aikman, 1989), thus LUE is greater at lower light intensities (Hirose and Bazzaz, 1998). To maintain the daily light integral (DLI) while decreasing the light intensity, the photoperiod must be extended. Therefore, implementing a longer photoperiod with an equivalent DLI may be able to increase photosynthetic efficiency and LUE, in turn improving crop growth and production. Previous research has shown the effectiveness of longer photoperiods to enhance crop growth using a variety of vegetables (Koontz and Prince, 1986; Soffe et al., 1977; Weaver and van Iersel, 2020) and strawberry (Tsuruyama and Shibuya, 2018). However, adjusting a crop’s photoperiod has variable effects depending on the crop species and cultivars. Hence, the impact of extended photoperiods on crop production should be studied on a species-specific basis, even considering individual cultivar. In certain regions with lower night electricity rates and monthly electricity delivery charge based on the electricity usage during the 1 peak hour in the month (the monthly electricity delivery charge is used for maintaining and building power grids, which is on the same scale of costs as electricity itself now, Ieso, 2025), longer supplemental light photoperiods with lower light intensity can also reduce the cost of electricity by reducing monthly electricity delivery charge and utilizing off-peak low-cost electricity during the night (Hao et al., 2022; Lanoue et al., 2021).

In theory, the greatest light fixture capital reduction, electrical cost savings, and crop growth benefits of long photoperiods should be observed during 24 hours of low intensity light [continuous lighting (CL)]. Previous research has recorded biomass enhancement of CL in lettuce (Kitaya et al., 1998; Ohtake et al., 2018), but CL-associated injury, characterized by interveinal leaf chlorosis, was observed in many other species including tomato and eggplant (Velez-Ramirez et al., 2011). Although the underlying mechanism of CL-injury in fruiting crops is still undetermined, Lanoue et al. (2019) were able to avoid CL-injury in greenhouse-grown tomato plants by alternating between red light during the day (06:00-18:00) and blue light at night (18:00-06:00). This research suggests that utilizing dynamic CL (i.e., a spectral and/or light intensity shift between the day and night) may be important for averting CL-injury and should be confirmed in other crops.

Greenhouse eggplant production is gaining popularity in northern high latitudes as growers attempt to diversify their crop portfolio and offset imported produce, especially throughout the winter. However, supplemental lighting research in greenhouse vegetable crops has mostly involved common greenhouse species like tomato, cucumber, and pepper (Demers and Gosselin, 2002). Some studies have targeted the effects of supplemental light quality on the biomass production in eggplant (Di et al., 2021; Dieleman et al., 2021), but a gap exists pertaining to the effects of supplemental light photoperiod on yield and leaf physiology in greenhouse-grown eggplant. Therefore, the objectives of this study were to 1) determine how extended supplemental photoperiods impact eggplant physiology and yield during the winter and 2) determine whether dynamic CL is a viable supplemental lighting strategy for greenhouse eggplant production. We hypothesized that the use of supplemental lighting will improve eggplant production, and the extension of supplemental light photoperiod at lower light intensity will improve light capture by enlarging leaf area and increasing photosynthetic efficiency. We also expected the use of blue light at night will avoid CL-associated plant injury.

## 2 Materials and methods

### 2.1 Plant material and growth conditions

Eggplant (*Solanum melongena*) seeds cv. Jaylo were sown on August 10, 2022, and seedlings were transplanted into rockwool cubes (Grodan Delta; Grodan, Roermond, Netherlands) on August 29, 2022. The eggplant seedlings were then transplanted onto rockwool slabs (Grodan Vital) on September 15, 2022, where they remained for the rest of the trial.

Two main stems from each plant were trained on a ‘V’ high-wire system at a plant density of 5.12 plants per m^2^ or 11.03 stems per m^2^. Adventitious stems (suckers) were pruned to support one fruit cluster per stem. Plants were drip-irrigated as needed with a complete nutrient solution [Ontario Ministry of Agriculture, Food and Rural Affairs (OMAFRA), 2010]. Irrigation setpoints began with an electrical conductivity (EC) of 2.5 dS m^-1^ and a pH of 5.8. The EC was increased to 3.0 dS m^-1^ one month after the initiation of supplemental light treatments to accommodate for the increase in fruit production. Greenhouse compartment temperature was regulated between 22-25°C during the day and 19-23°C at night via hot water heat pipes and roof vents. The relative humidity of each compartment ranged from 60-75%. CO_2_ was sustained at 800 ppm while the vents were closed and 400 ppm while the vents were open.

### 2.2 Supplemental light treatments and greenhouse setup

This trial was performed in two adjacent 50 m^2^ double-layer polyethylene greenhouse compartments at the Harrow Research and Development Centre (Agriculture and Agri-Food Canada, Harrow, ON, Canada; 42.03°N, 82.9°W). The greenhouse compartments were each divided into four sections using light abatement curtains (Obscura 9950 FR W; Ludvig Svensson, Kinna, Sweden) that blocked light transmission between treatments but allowed for ambient flow of air and moisture. The curtains remained closed during the night and on cloudy days when the outdoor radiation was less than 400 W m^-2^ and were opened on sunny days or when the outdoor radiation was greater than or equal to 400 W m^-2^.

**Figure 1:**
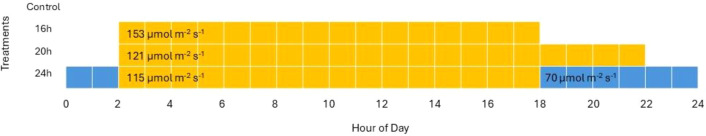
Schematic of supplemental light treatments. Experimental photoperiod treatments are represented by Control, 16-hour, 20-hour, 24-hour. Bars filled in yellow denote a white light spectrum and bars filled in blue denote a blue light spectrum.

**Figure 2:**
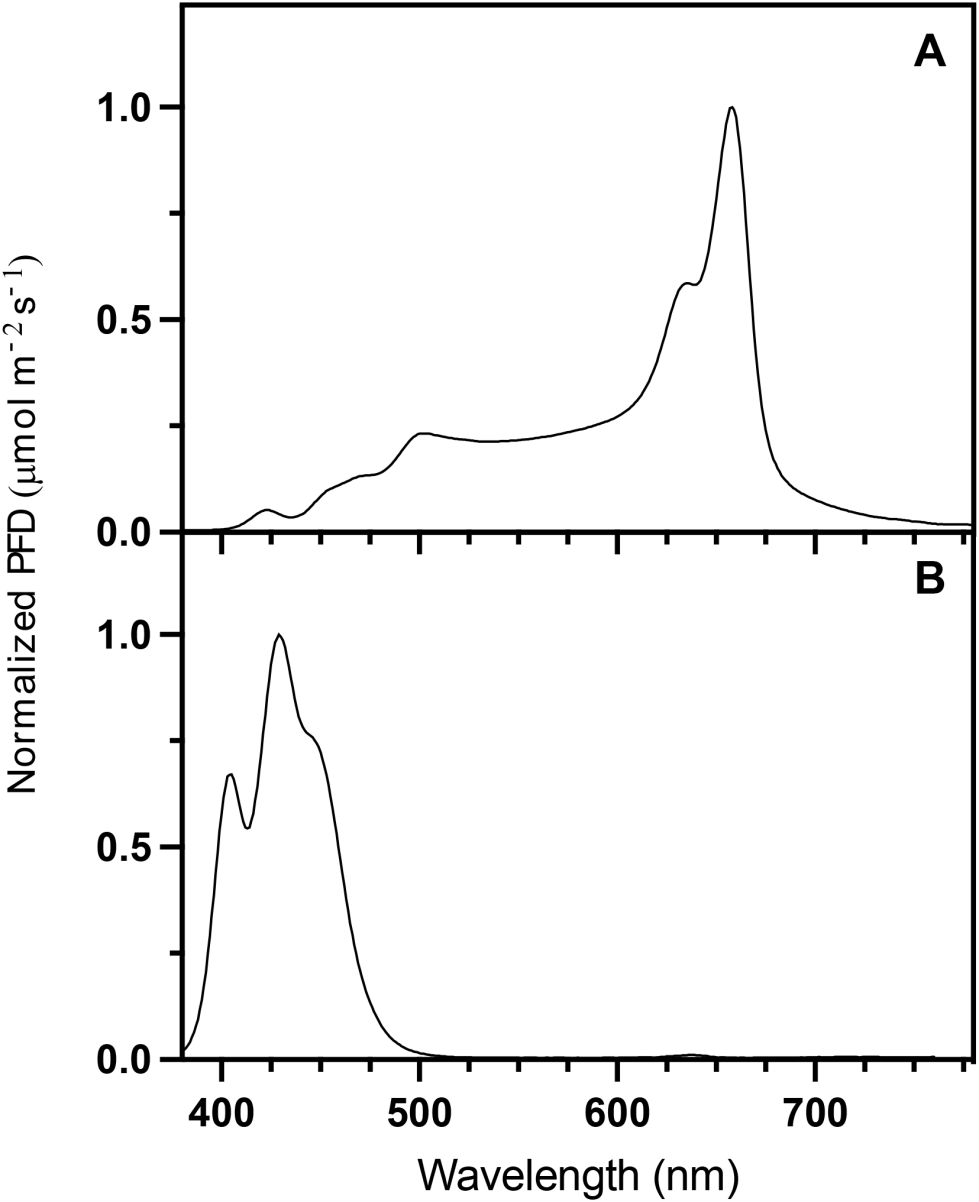
Normalized spectral photon flux density (PFD) distribution (380–780 nm) of supplemental white light **(A)** and blue light **(B)** treatments using Sollum LED top lights.

Four supplemental light treatments were randomly assigned to each quadrant of the greenhouse compartments. The treatments consisted of four different supplemental photoperiods: no supplemental light (control), 16 hours of broad-spectrum white light (16-hour), 20 hours of broad-spectrum white light (20-hour), and 16 hours of broad-spectrum white light followed by 8 hours of narrowband blue light (24-hour) (**Figure 1**). Broad-spectrum white light had a spectral composition of 12% blue (400–499 nm), 30% green (500–599 nm), 56% red (600–699 nm), and 2% far-red (700–780 nm) (**Figure 2A**). The blue light mostly consisted of wavelengths 400–499 nm (**Figure 2B**).

Excluding the control, each treatment’s supplemental daily light integral (DLI) was 8.64 mol m^-2^ day^-1^ of photosynthetically active radiation (PAR). To balance the DLI for all treatments the light intensities of longer photoperiods were dimmed. The supplemental PAR provided to each treatment were 153 ± 3 µmol m^-2^ s^-1^ (white light), 121 ± 2 µmol m^-2^ s^-1^ (white light), and 115 ± 3 µmol m^-2^ s^-1^ (white light) followed by 70 ± 2 µmol m^-2^ s^-1^ (blue light) for the 16-hour, 20-hour, and 24-hour treatments, respectively. The 20-hour and 24-hour treatments will reduce the electricity monthly delivery charge by 21 and 25%, respectively, in comparison to the 16-hour treatment, because monthly electricity delivery charge is based on the electricity usage during the 1 peak hour in each month (Ieso, 2025). Supplemental light treatments began on November 7, 2022 (53 days after transplanting), using dynamic LED top lights (SF04; Sollum Technologies Inc., Montreal, QC, Canada). The use of a blue light during the night was selected because our prior study (Lanoue et al., 2019) and others (Velez-Ramirez et al., 2017) elucidated that dim blue light during the night period could reduce or eliminate CL injury. Average supplemental PAR at the apex of the crop canopy was measured once per month at night using a line quantum sensor (LI-191R; LI-COR Biosciences, Lincoln, NE, USA), and the light fixtures were dimmed to ensure each treatment received the target intensity of light.

### 2.3 Plant morphology measurements

Four random plants from each treatment (n=4) were destructively harvested for morphology measurements 67 and 126 days after the start of light treatments (DAL). Main stem length, stem diameter, number of leaves, number of nodes, leaf fresh weight, and stem fresh weight were measured on both main stems of each sample plant. Total leaf area of each plant was also recorded using a LI-COR leaf area machine (LI-3100C; LI-COR Biosciences). Leaves and stems were then dried in a 75°C oven for 7 days when a consistent sample weight was recorded. The dry weight of stems and leaves was summed as total plant dry mass. The dry matter weight of leaves and stems was then divided by the fresh weight of both leaves and stems to calculate stem and leaf water content. Harvest index was also calculated to estimate the generative balance of the plants by dividing the average total fruit yield per main stem by the average fresh weight of each main stem.

### 2.4 Light response curves and chlorophyll fluorescence

Leaf light response curves (LRC) [net carbon exchange rate (NCER) responses to light intensity] were produced within two days on four occasions: once per month from December 2022 to March 2023. LRCs were generated from single leaves using a portable photosynthesis system (LI-6800; LI-COR Biosciences). Three LRCs were produced per treatment using leaves within the third node from the apex of the plants. The LI-6800 leaf chamber conditions were set to the following: CO_2_ of 1000 μL L^-1^, block temperature of 24°C, relative humidity of 65%, and light intensities of 1500, 1000, 750, 500, 250, 100, 75, 50, 25, 10, 0 μmol m^-2^ s^-1^. Before producing each LRC, each leaf was placed into the LI-6800 chamber with a light intensity of 1500 μmol m^-2^ s^-1^, and the LRC only began once a steady NCER was observed. While generating the LRC, measurements were recorded when a steady NCER was observed at each light intensity. Light-adapted chlorophyll fluorescence was also measured between each step in light intensity using a high-intensity flash (6000 μmol m^-2^ s^-1^ rectangular flash for 0.8 s). Following the LRC, each leaf was kept in the dark chamber for an additional 10 minutes, and another high-intensity flash was used to calculate dark-adapted chlorophyll fluorescence (F_v_/F_m_). NCER was plotted against light intensity (PPFD) and fitted to an asymptotic regression model Y= a + b*e^(C*X)^ using Prism (Version 9.5; Graphpad Software, San Diego, CA, USA) where Y, X, a, and *e* represent NCER, PPFD, light saturated NCER (A_max_), and Euler’s constant, respectively (Rodriguez-Morrison et al., 2021). The light saturation points (LSP) were calculated by determining the PPFD within the LRC that corresponded with 95% of the A_max_. A linear regression was plotted using the measured NCER at 0, 10, and 25 µmol m^-2^ s^-1^ to calculate light compensation point (LCP) and quantum yield (QY). LCP is equal to the PPFD at which net-zero NCER (x-intercept) was achieved. QY is equal to the slope of the line, which is the molar ratio between net carbon assimilated and photons absorbed (Φ).

### 2.5 Fruit harvest

Eggplant fruit was harvested as “miniature eggplant” and each fruit was picked when they reached approximately 200 g. The average fruit weight in each treatment was 201 ± 9 g, 204 ± 9 g, 197 ± 9 g, and 211 ± 9 g in the control, 16-hour, 20-hour, and 24-hour treatment, respectively. Fruit was bulk harvested three days per week beginning October 19, 2022. Once light treatments began, fruit continued to be harvested three times per week, but the yield of each treatment was separated and weighed accordingly.

### 2.6 Statistical analysis

Statistical analyses on average total yield plant^-1^, total plant dry mass, plant morphology, LRC parameters, and Fv/Fm were performed using R (RStudio, Version 1.4.1717) with a one-way analysis of variance (ANOVA) followed by Tukey’s multiple comparisons test with a single pooled variance to assess for significant (α=0.05) differences among photoperiod treatments. All data were checked for normality, homogeneity, and heteroskedasticity.

## 3 Results

### 3.1 Fruit yield

**Figure 3:**
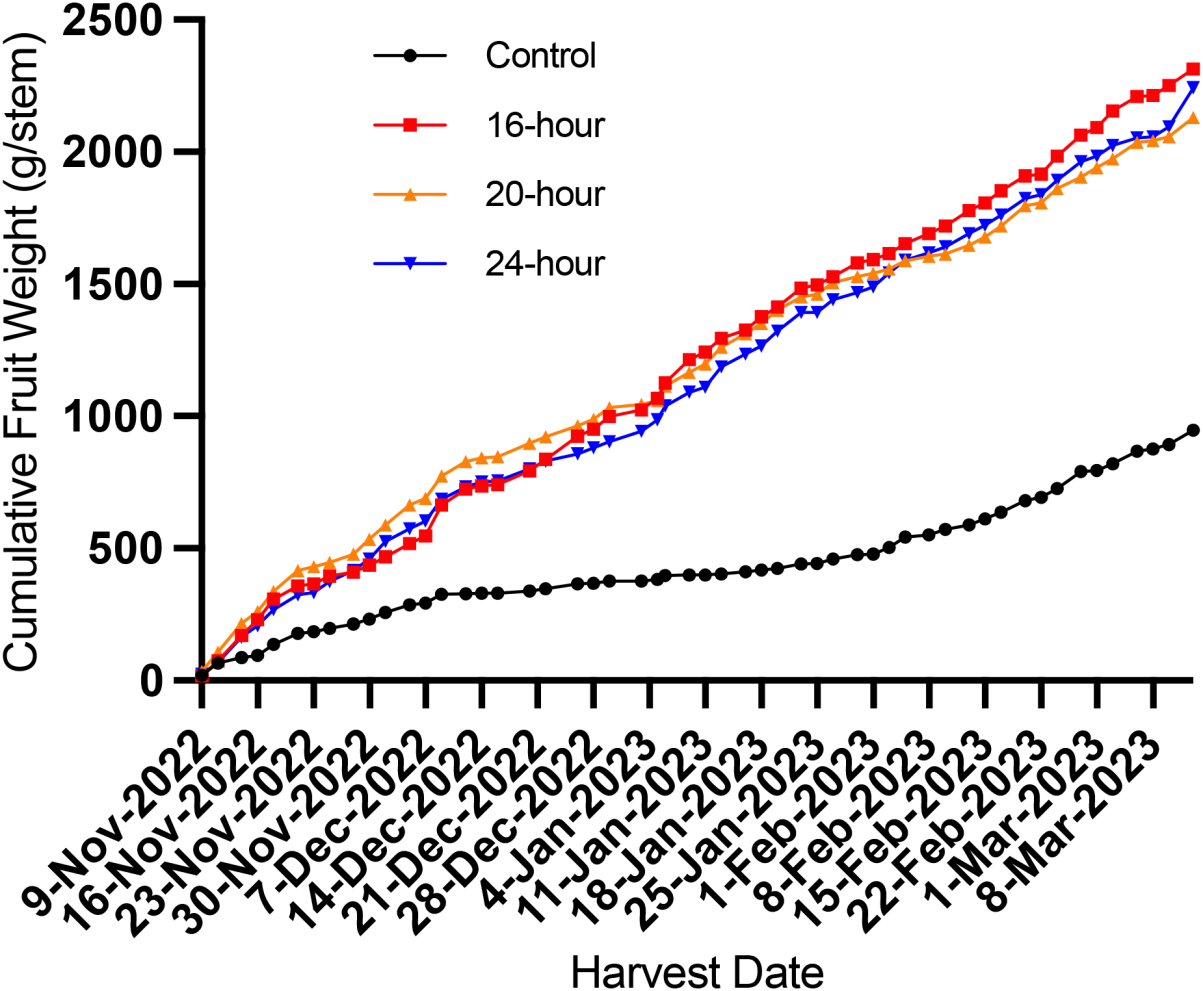
Cumulative harvested *Solanum melongena* (eggplant) cv. Jaylo fruit weight per main stem of different supplemental light photoperiod treatments. No supplemental light (control), 16-hour, 20-hour, and 24-hour supplemental light treatments began on November 7, 2022.

All supplemental light treatments significantly increased the total fruit yield (per stem and yield per m^2^ basis) of winter-grown eggplant compared to an unlit control group (**Figure 3**; **Table 1**). The 16-hour, 20-hour, and 24-hour supplemental light treatments increased total eggplant yield by 144%, 124%, and 135% compared to the unlit control treatment (P<0.05), respectively (**Table 1**). Although the total yields amongst the supplemental light treatment groups were not significantly different (p=0.53), it should be noted that some treatments exhibited marginally greater yields at various periods throughout the production cycle. The plants treated with 20-hour of supplemental light appeared to have improved yield between 14–42 DAL with fruit production that subsided for the remainder of the growth cycle relative to the other lit treatments (**Figure 3**). Plants treated with 24-hour of supplemental light appear to have a fruit production trend similar to the 16-hour treatment (**Figure 3**). While plants grown under 20-hour of supplemental light had similar yield throughout the study as both 16-hour and 24-hour plants, yield tended to be lower at the latter periods of production in the 20-hour treatment.

**Table 1:** Total yield per main stem or per m^2^ of *Solanum melongena* (eggplant) cv. Jaylo under different supplemental light photoperiod treatments recorded between Nov. 27, 2022 - March 8, 2023 (119 DAL).

Light treatment	Control	16-Hour	20-Hour	24-Hour
Total Yield per stem (kg stem^-1^)	0.95 ± 0.07^b^	2.30 ± 0.06^a^	2.12 ± 0.17^a^	2.22 ± 0.08^a^
Total Yield per m^-2^ (kg m^-2^)	4.91 ± 0.41^b^	11.66 ± 0.67^a^	10.92 ± 0.66^a^	11.62 ± 0.37^a^

Total yield was averaged across four rows of plants (n = 4) ± standard error. Data with the same lowercase letters were not significantly different (p<0.05).

### 3.2 Plant morphology

**Figure 4:**
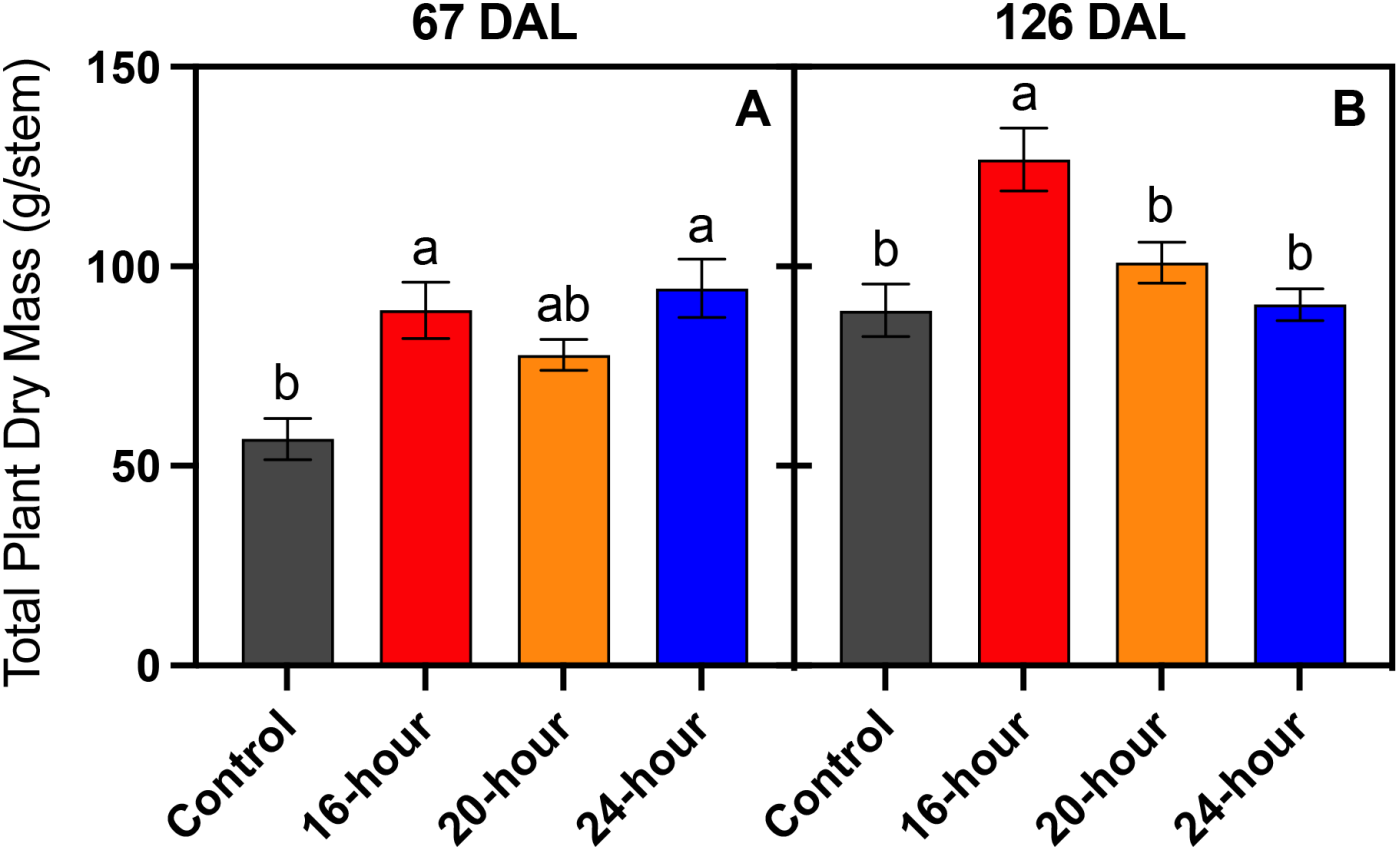
Cumulative leaf and stem dry mass per main stem of *Solanum melongena* (eggplant) cv. Jaylo plants taken 67 days **(A)** and 126 days **(B)** after the start of different supplemental light photoperiods treatments. Control, no supplemental light; 16-hour, 20-hour, and 24-hour indicate the number of hours per day with supplemental light. Bars represent means ± standard error (n = 4). Data bearing the same lowercase letters were not significantly different within each graph frame (*p* < 0.05).

At 67 DAL, the control plants had the lowest total plant dry mass, the 16-hour and 24-hour treatments had the greatest total plant dry mass, and the 20-hour treatment contained a total plant dry mass that was non-significant from the control, 16-hour, or 24-hour treatments (**Figure 4A**). At 126 DAL, plants grown under the 16-hour treatment had the largest plants compared to plants grown under the other treatments (**Figure 4B**). Interestingly, plants grown under the no supplemental light control had similar total plant dry mass (excluding fruit and root dry matter) as plants from the 20-hour and 24-hour treatments. At 126 DAL plants in all treatments had similar stem length and diameter, but the number of leaves, leaf area, and leaf fresh weight were greater in the control and 16-hour treatments (**Table 2**). Plants in the control had the lowest leaf and stem water contents on both 67 and 126 DAL (**Table 2**).

**Table 2:** Plant morphology attributes of *Solanum melongena* (eggplant) cv. Jaylo plants grown under different supplemental light photoperiods (Control, no supplemental light; 16-hour, 20-hour, and 24-hour supplemental light).

Time of measurement	67 DAL				126 DAL			
Treatment	Control	16-Hour	20-Hour	24-Hour	Control	16-Hour	20-Hour	24-Hour
Main Stem Length (cm)	141.3 ± 7.3^a^	145.5 ± 3.8^a^	145.1 ± 5.3^a^	152.6 ± 5.2^a^	186.9 ± 4.1^a^	192.0 ± 4.5^a^	199.6 ± 5.3^a^	199.6 ± 5.3^a^
Stem Diameter (mm)	6.8 ± 0.3^a^	7.5 ± 0.5^a^	7.7 ± 0.2^a^	7.3 ± 0.3^a^	4.9 ± 0.3^a^	5.9 ± 0.3^a^	5.7 ± 0.2^a^	5.7 ± 0.3^a^
Number of Leaves	43.8 ± 2.7^a^	51.0 ± 2.4^a^	51.4 ± 2.4^a^	57.9 ± 2.9^a^	53.9 ± 3.3^a^	55.3 ± 2.3^a^	35.6 ± 2.4^b^	32.4 ± 3.1^b^
Number of Nodes	12.0 ± 0.4^a^	12.8 ± 0.3^a^	12.6 ± 0.3^a^	13.1 ± 0.3^a^	19.4 ± 0.4^b^	20.6 ± 0.5^ab^	21.4 ± 0.3^a^	20.5 ± 0.5^ab^
Leaf Area (m²)	1.12 ± 0.09^a^	1.32 ± 0.07^a^	1.11 ± 0.06^a^	1.38 ± 0.13^a^	1.19 ± 0.09^a^	1.13 ± 0.09^a^	0.69 ± 0.09^b^	0.6 ± 0.07^b^
Leaf Fresh Weight (g)	260.5 ± 23.8^a^	340.8 ± 20.9^ab^	278.2 ± 15.3^ab^	354.6 ± 34.0^a^	266.8 ± 22.2^ab^	294.9 ± 27.4^a^	189.8 ± 25.4^bc^	156.7 ± 16.6^c^
Leaf Dry Weight (g)	26.2 ± 2.4^b^	42.8 ± 3.4^a^	34.5 ± 1.7^ab^	43.7 ± 3.5^a^	39.3 ± 2.8^b^	52.3 ± 3.1^a^	34.7 ± 3.5^bc^	24.9 ± 2.6^c^
Stem Fresh Weight (g)	246.1 ± 24.4^b^	324.6 ± 25.7^ab^	291.4 ± 19.5^ab^	343.7 ± 25.3^a^	305.4 ± 23.1^b^	399.0 ± 26.7^a^	344.9 ± 16.2^ab^	346.5 ± 14.9^ab^
Stem Dry Weight (g)	30.6 ± 3.0^b^	46.2 ± 3.8^a^	43.3 ± 2.8^ab^	50.8 ± 4.0^a^	49.7 ± 3.9^b^	67.8 ± 5.3^a^	66.3 ± 2.0^a^	66.5 ± 3.3^a^
Leaf Water Content (%)	10.0 ± 0.2^b^	12.5 ± 0.3^a^	12.4 ± 0.4^a^	12.4 ± 0.4^a^	14.9 ± 0.4^c^	18.2 ± 0.8^ab^	18.8 ± 0.6^a^	15.9 ± 0.4^bc^
Stem Water Content (%)	12.4 ± 0.2^b^	14.2 ± 0.3^a^	14.8 ± 0.3^a^	14.8 ± 0.2^a^	16.3 ± 0.3^b^	18.6 ± 0.4^a^	19.3 ± 0.5^a^	18.9 ± 0.3^a^
Harvest Index (%)	65.6 ± 8.5^c^	151.7 ± 2.2^ab^	169.7 ± 12.6^a^	129.8 ± 4.4^b^	134.8 ± 11.0^c^	274.2 ± 7.5^b^	328.4 ± 24.7^ab^	365.5 ± 12.6^a^

Data are mean ± standard error (n = 4). Data bearing the same lowercase letters were not significantly different within the time of measurement and row (*p* < 0.05).

### 3.3 Light response curves and chlorophyll fluorescence

**Figure 5:**
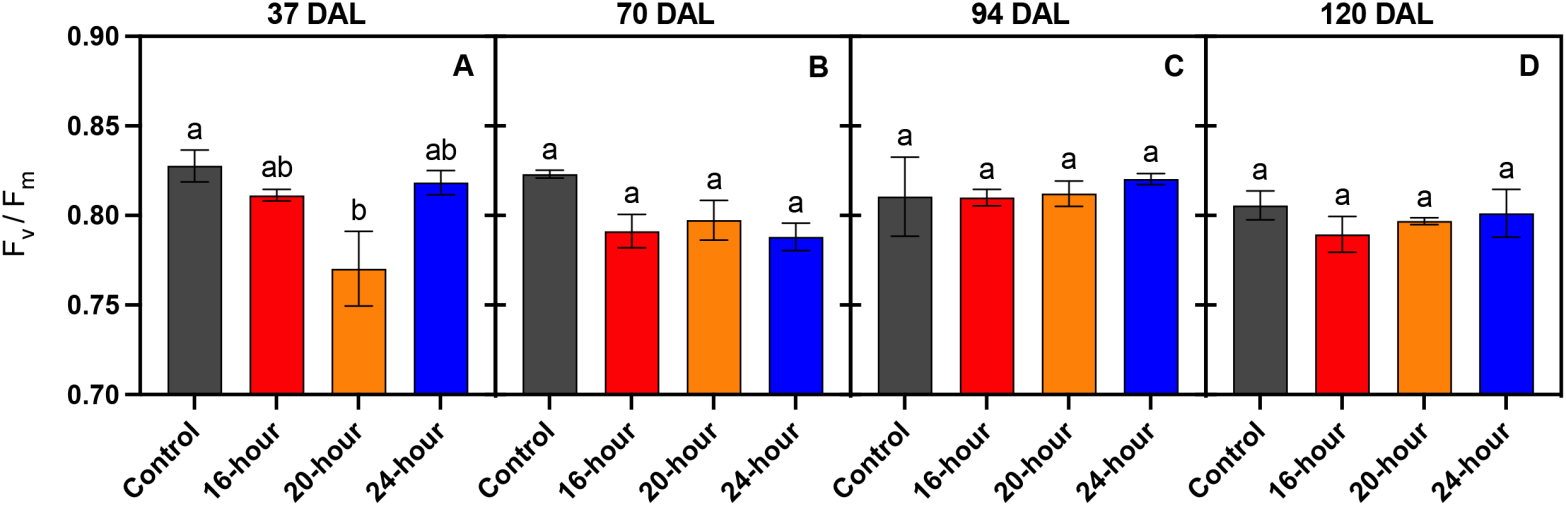
Dark-adapted chlorophyll fluorescence (Fv/Fm) of leaves of *Solanum melongena* (eggplant) cv. ‘Jaylo’ plants grown under different supplemental light photoperiods (Control, no supplemental light; 16-hour, 20-hour, and 24-hour). Each graph represents measurements repeated 37, 70, 94, and 120 DAL respectively. Bars represent mean ± standard error (n = 3). Data bearing the same lowercase letters within each given panel were not significantly different (*p* < 0.05).

The maximum quantum efficiency of PSII photochemistry (F_v_/F_m_) is often used to monitor leaf stress through the measurement of photo-inhibition. Leaf stress under a particular environmental factor, in this case photoperiod, corresponds with a lower F_v_/F_m_ value. After measuring dark adapted chlorophyll fluorescence early in the study (37 DAT), leaves under the 20-hour treatment were observed to have the lowest F_v_/F_m_ values, indicating the highest amount of stress (**Figure 5A**). During this same period, no difference in leaf stress was apparent in the 16-hour or 24-hour light treatment compared to the no light control. In subsequent measurements (70–120 DAT), as the natural light intensity increasingly played a larger role in overall DLI, no difference in F_v_/F_m_ was observed in leaves (**Figures 5B–D**).

**Figure 6:**
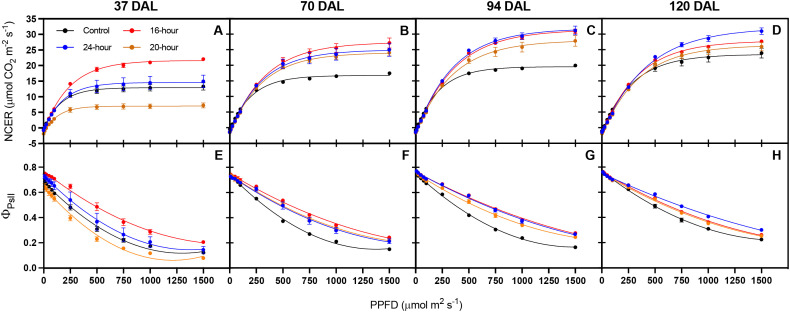
Net carbon exchange rate (NCER) **(A–D)** and maximum quantum yield of photosystem II (Φ_PsII_) **(E–H)** of *Solanum melongena* (eggplant) cv. Jaylo leaves under different supplemental light photoperiod treatments. Each graph represents measurements conducted 37, 70, 94, and 120 DAL, respectively. Individual data points represent the mean ± standard error (n = 3). A line is shown when the regression is significant (*p* < 0.05).

**Figure 6** demonstrates how the plants’ leaves in each treatment physiologically responded to various supplemental light photoperiods. It was apparent that the plants’ leaves had more distinctive responses to the supplemental photoperiod treatments during the first set of physiological measurements (**Figures 6A, E**). However, as the experiment progressed, the physiological response to light was similar under all lit treatments (**Figure 6**). After the first set of leaf physiology measurements on 37 DAL, the 20-hour treatment tended to develop leaves with lower net carbon exchange rate (NCER) and quantum yield of PSII in the light adapted state (Φ_PsII_) at high light intensities (1000-1500 μmol m^-2^ s^-1^) (**Figures 6A, E**). Conversely, the 16-hour group tended to develop leaves with the greatest NCER and Φ_PsII_ (**Figures 6A, E**).

**Figure 7:**
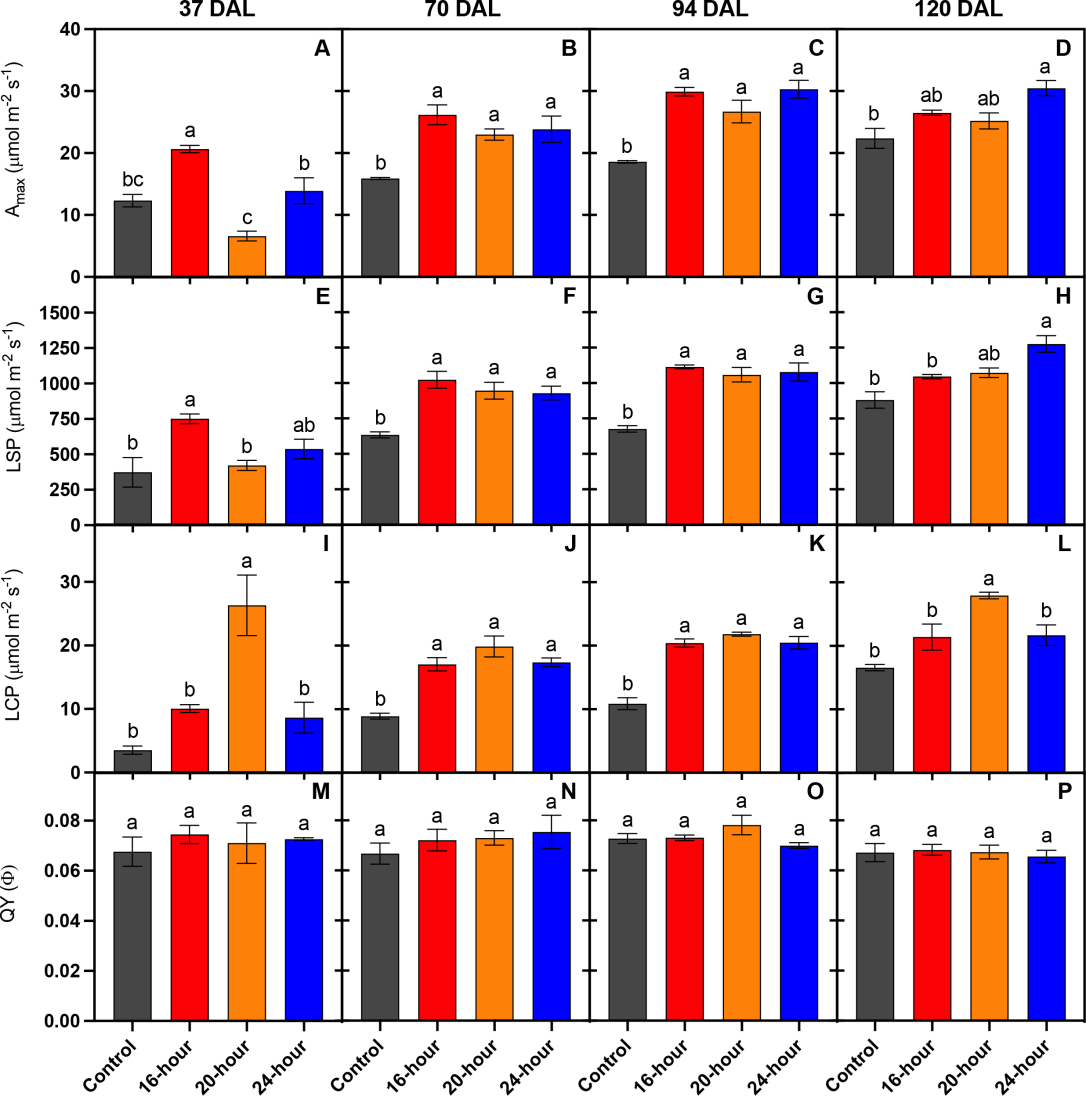
Light-saturated net carbon exchange rate (A_max_) **(A–D)**, light saturation point (LSP) **(E–H)**, light compensation point (LCP) **(I–L)**, and quantum yield (QY) **(M–P)** of leaves within the canopies of *Solanum melongena* cv. Jaylo plants grown under different supplemental light photoperiods. Each graph represents measurements repeated 37, 70, 94, and 120 DAL respectively. Bar is mean ± standard error (n = 3). Data bearing the same lowercase letters were not significantly different within each graph frame (*p* < 0.05).

**Figure 7** represents the parameters related to the light response curves generated in **Figures 6A–D**. After 37 DAL the plants treated with a 16-hour supplemental photoperiod had leaves with the greatest capacity to assimilate carbon (A_max_), there was no significant difference between the control and the 20-hour or 24-hour treatments, but the 24-hour treatment had significantly greater A_max_ values compared to the 20-hour treatment (**Figure 7A**). The 16-hour treatment had leaves with the highest LSP, while the control and 20-hour groups had leaves with significantly lower LSPs (**Figure 7B**). The highest LCP was found within the 20-hour treated leaves, and QY was not significantly affected by any treatment (**Figures 7C, D**). In subsequent measurements, the 24-hour treatment contained the greatest LSP (**Figure 7H**) and the 16-hour treatment group contained the greatest LCP (**Figure 7L**). However, after the first period (37 DAL) of leaf physiology measurements, no significant difference existed among the lit treatments between A_max_ and QY (**Figures 7B–D, N–P**).

## 4 Discussion

Carbon assimilation, plant biomass, and fruit yield during winter production of greenhouse vegetables are often limited by the total amount of light available to the crop. In general, supplemental lights aid in the growth and yield of the crop by maintaining a consistently elevated DLI (intensity x photoperiod) throughout the winter. It is apparent that plants’ LUE decreases as light intensity increases since the process of assimilating carbon is less efficient and can demonstrate signs of inefficiency and potentially damage at elevated light intensities (Fan et al., 2013). Extending photoperiod at lower light intensity can therefore be used as an economic lighting strategy to sustain DLI throughout the winter. However, some crops display signs of injury when the photoperiod was extended beyond a threshold (Hillman, 1956; Velez-Ramirez et al., 2011). Specifically, eggplant grown under continuous lighting at 300 µmol m^-2^ s^-1^ in a growth chamber showed reduced photosynthetic rates and reduced dark adapted chlorophyll fluorescence readings indicating injury (Shibaeva et al., 2024).

The total observed fruit yield from lit treatments (16-hour, 20-hour, and 24-hour) were 124-144% higher than the non-lit control, which further proves the effectiveness of supplemental lighting for winter production of greenhouse vegetables in northern high latitudes, specifically in eggplant. All lit treatments displayed no significant differences in total fruit yield, but the 20-hour treatment tended to yield slightly more fruit early in the trial (14–42 DAL). Therefore, extending the supplemental photoperiod had no significant impact on total fruit yield, but a 20-hour photoperiod may be utilized to encourage early fruit production. However, the increase in early production from the 20-hour treatment corresponded with early signs of plant light stress. **Figures 6A, E**, and **Figures 7A, E, I, M** demonstrate the apparent damage to photosynthetic machinery within the 20-hour treatment after 37 DAL. The 20-hour treatment produced leaves with the lowest Amax, the highest LCP, and tended to present lower NCER, Φ_PSII_, NPQ, and ETR values (**Supplementary Figure S1**), especially at higher light intensities. The decreased photosynthetic efficiency, often correlated with CL-associated injury, was not observed in the second leaf physiology measurements taken at 70 DAL and could explain why we did not detect a significant difference in many initial plant morphology measurements at 67 DAL (Matsuda et al., 2014). We did, however, record the greatest harvest index within the 20-hour treatment group after 67 DAL, alluding to more generative plants, reflected by increased fruit production, and slightly decreased vegetative biomass stimulated by the 20-hour supplemental light treatment. It is important to note that many *Solanaceae* species are day-neutral, while some *Solanaceae* species flower more rapidly under long or short photoperiods (Soyk et al., 2017). Therefore, the increased generative response could be a direct response to the longer white light photoperiod. However, this increased generative response could also be indicative of increased light stress; and therefore, an indirect response to a longer white light photoperiod.

As the control plants received less light compared to the lit groups, the lower harvest index in the control plants can be attributed to a reduction in fruit yield rather than an increase in vegetative biomass. In contrast, the harvest index after 126 DAL was greatest in the 20-hour and 24-hour treatments, not because there was an increase in fruit production, but largely due to the considerable decrease in total plant dry mass. The decreased plant dry mass among the 20-hour and 24-hour treatments observed late in the trial (126 DAL) may be indicative of a photoperiod-induced response; however, this response is likely unrelated to leaf light stress, as leaves within all lit treatments tended to display similar physiological responses to light at 120 DAL (**Figure 5D**, **Figures 6D, H**).

We hypothesized that there would be a greater vegetative biomass in plants as the supplemental photoperiod increased, akin to previous research regarding the effects of photoperiod extension (Koontz and Prince, 1986; Soffe et al., 1977; Weaver and van Iersel, 2020). Specifically, we expected to invoke a shade avoidance response, observed through greater leaf areas and leaf dry weight among the longer photoperiod treatments due to a lower supplemental PPFD. However, our results did not display a significant increase in plant biomass beyond the 16-hour supplemental light photoperiod which is in contrast to results published by Agrawal et al. (2025). A notable difference between the two studies is Agrawal et al. (2025) increased the overall DLI by adding additional light during the night period whereas our DLI was constant among the 16, 20, and 24-hour treatments. When normalizing for DLI, the results presented here could be explained through a variety of species-specific responses to photoperiod. For instance, lettuce has shown to increase leaf area and dry biomass with extended photoperiods (Weaver and van Iersel, 2020) but during its growth, developing leaves are the only form of sink tissue. While crops like eggplant and day-neutral strawberry must allocate carbon to developing leaves, as well as flowers and fruit (Heide, 1977). Therefore, a more complex relationship may exist within day-neutral crops that prohibits leaf expansion and should be researched further.

Previous studies have avoided continuous lighting-associated plant injury by utilizing an alternating spectrum throughout the day and night lighting (Lanoue et al., 2019; Ohtake et al., 2018), but plant injury was quantified through plant morphology, LRCs, and dark-adapted chlorophyll fluorescence. These parameters are useful but exclude factors pertaining to light-adapted chlorophyll fluorescence like Φ_PSII_, NPQ, and ETR, which may prove to be a more accurate depiction of leaf stress or damage. Therefore, there might have been leaf light stress presented in previous studies which was overlooked due to the plant injury metrics used. In the present study, we found that alternating between supplemental white light during the day and blue light at night was able to avoid reductions in fruit yield while sustaining plant growth similar to the 16-hour treatment. Although plants did not display damage through morphological traits, the plants treated with 24-hour of supplemental light for 37 days exhibited a lower A_max_ and tended to display lower Φ_PSII_ and ETR values (**Supplementary Figure S1**), which signifies some breakdown of photosynthetic machinery. It should be noted that all plants within the lit treatment groups acclimated to the extended photoperiods and all negative responses subsided two months following the induction of supplemental light treatments.

Dark-adapted chlorophyll fluorescence measurements (i.e., the measurement of maximum quantum efficiency of PSII) have long been the standard to assess leaf stress status (Baker, 2008). Specifically, within photoperiodic research, the measurement of F_v_/F_m_ has been used to identify stress associated with extended photoperiods (Haque et al., 2015; Lanoue et al., 2019; Velez-Ramirez et al., 2014). However, recent evidence suggests that the use of dark-adapted chlorophyll fluorescence may not be the most appropriate measurement to assess the ability of a leaf to utilize light within photosynthesis (Lanoue et al., Unpublished/Under Review). Dark adapted chlorophyll fluorescence measurements assess the state of PSII under theoretical conditions where all reaction centers are open. On the other hand, light adapted chlorophyll fluorescence assesses the ability of PSII to absorb/utilize light when already exposed to light – more in line with typical daytime conditions. In **Figures 5B, C**, F_v_/F_m_ is not significantly different between leaves under all the treatments, indicating that no treatment has increased stress compared to the no light control. However, it is clear, through light adapted chlorophyll fluorescence measurements, the operating efficiency of PSII (**Figures 6F, G**) was reduced in the control plants under actinic light level above 100 µmol m^-2^ s^-1^. Consequently, the NCER of leaves under the no light control were reduced above this light level compared to all leaves exposed to light (**Figures 7B, C**).

Leaves under only natural light are expected to have no injury/stress related to the light environment. However, the absence of stress indicators does not mean the leaf is optimally primed for photosynthesis. While the process of photosynthetic acclimation is complicated, the first step in optimizing photosynthetic efficiency is adequate light capture. Therefore, exposing leaves to supplemental light that does not cause injury has primed the photosynthetic machinery of eggplant leaves to better utilize higher light intensities, ideal for adapting to naturally fluctuating light environments (Lichtenthaler et al., 1981). The efficiency of light capture and utilization in the photosynthetic process is further observed by the lower levels of NPQ in leaves exposed to supplemental light compared to the control (**Supplementary Figure S1**). Taken together, this data shows a disconnect between dark adapted and light adapted chlorophyll fluorescence data. Therefore, we caution the sole use of dark-adapted chlorophyll fluorescence to interpret the stress and photosynthetic status of leaves. Instead, we believe that coupling dark and light adapted measurements allows for a better understanding of whether stress is the cause of photosynthetic/biomass reduction, or if reduced light-use-efficiency is due to a lack of photosynthetic acclimation.

In practice, the utilization of light-adapted chlorophyll fluorescence measurements can provide more practical information than dark-adapted measurements. For example, a reduction in Φ_PSI_ could indicate that increasing the light intensity would not have appreciable gains in photosynthesis (observed as a reduction in light-use-efficiency) and could ultimately harm the plant. The disconnect between light- and dark-adapted measurements observed in this study details this importance. If dark-adapted measurements were the only ones taken, due to the similarities between F_v_/F_m_ observed later in the study (**Figures 5B–D**), a conclusion would be made that plants under all treatments would react to an increase in light intensity in the same fashion. However, this was clearly not the case as indicated by NCER curves (**Figures 6B–D**) where plants under the control treatment with no supplemental light struggled to utilize higher light intensities. This struggle to utilize additional light was only observed via light-adapted measurements. A light-adapted measurement can therefore be used as a tool to interpret real-time feedback from the plant in order to control the supplemental light intensity. Future research should focus on such bio-feedback lighting mechanism.

## 5 Conclusion

In summary, this study highlighted the impact of extended supplemental light photoperiods on yield, morphology, and leaf physiology of greenhouse eggplant. The findings in this study provide evidence to support the use of a dynamic CL strategy for winter eggplant cultivation in northern high latitudes since no significant difference was found between the yield or leaf physiology of plants treated with 16-hour and 24-hour of supplemental light. Remarkably, all plants treated with supplemental light exhibited similar yields, and increased total yield by 124-144% compared to the unlit control. The greatest level of leaf stress was recorded 37 DAL within the 20-hour group, but all physiological parameters recovered as time progressed. We suggest that future research regarding leaf photosynthetic efficiency should utilize both dark-adapted and light-adapted fluorescence metrics for a more precise depiction of plant stress. Future studies on greenhouse eggplant should also explore the ramifications of higher DLIs through increasing supplemental light intensity to possibly achieve greater plant responses.

## Data Availability

The original contributions presented in the study are included in the article/**Supplementary Material**. Further inquiries can be directed to the corresponding author.

## Supplementary material

Supplementary Figure 1 Nonphotochemical chlorophyll fluorescence quenching (NPQ) **(A–D)** and electron transport rate (ETR) **(E–H)** of *Solanum melongena* (eggplant) cv. Jaylo leaves under four different supplemental light photoperiod treatments. Each graph represents measurements repeated 37, 70, 94, and 120 DAL respectively. Individual data points represent the mean of three replicate leaves (n = 3) and the error bars represent ± standard error. A line is shown when the regression is significant (*p* < 0.05).
