# Supplementary material for: Growth and yield of greenhouse eggplant under extended photoperiods using light emitting diodes
Source: Front Plant Sci. 2026 Jan 19;16:1737061. doi: 10.3389/fpls.2025.1737061 (PMC12861898; doi:10.3389/fpls.2025.1737061)
Supplement: Supplementary Figure 1 — Nonphotochemical chlorophyll fluorescence quenching (NPQ) (A–D) and electron transport rate (ETR) (E–H) of Solanum melongena (eggplant) cv. Jaylo leaves under four different supplemental light photoperiod treatments. Each graph represents measurements repeated 37, 70, 94, and 120 DAL respectively. Individual data points represent the mean of three replicate leaves (n = 3) and the error bars represent ± standard error. A line is shown when the regression is significant (p < 0.05). [file DataSheet1.docx]

**Supplementary Figure 1.** Nonphotochemical chlorophyll fluorescence quenching (NPQ) (A, B, C, D) and electron transport rate (ETR) (E, F, G, H) of *Solanum melongena* (eggplant) cv. Jaylo leaves under four different supplemental light photoperiod treatments. Each graph represents measurements repeated 37, 70, 94, and 120 DAL respectively. Individual data points represent the mean of three replicate leaves (n = 3) and the error bars represent ± standard error. A line is shown when the regression is significant (*p*<0.05).
